# Association of body mass index and cardiotoxicity related to anthracyclines and trastuzumab in early breast cancer: French CANTO cohort study

**DOI:** 10.1371/journal.pmed.1002989

**Published:** 2019-12-23

**Authors:** Elisé G. Kaboré, Charles Guenancia, Ines Vaz-Luis, Antonio Di Meglio, Barbara Pistilli, Charles Coutant, Paul Cottu, Anne Lesur, Thierry Petit, Florence Dalenc, Philippe Rouanet, Antoine Arnaud, Olivier Arsene, Mahmoud Ibrahim, Johanna Wassermann, Geneviève Boileau-Jolimoy, Anne-Laure Martin, Jérôme Lemonnier, Fabrice André, Patrick Arveux

**Affiliations:** 1 “Health across Generations” Team, Inserm U1018, Centre for Research in Epidemiology and Population Health (CESP), Villejuif, France; 2 Cardiology Department, University Hospital Dijon Bourgogne, Dijon, France; 3 Institut Gustave Roussy, Villejuif, France; 4 Centre Georges-François Leclerc, Dijon, France; 5 Institut Curie, Paris, France; 6 Centre Alexis Vautrin, Vandoeuvre les Nancy, France; 7 Centre Paul Strauss, Strasbourg, France; 8 Institut Claudius Regaud, Toulouse, France; 9 Institut du Cancer de Montpellier, Montpellier, France; 10 Clinique Sainte Catherine, Avignon, France; 11 Centre Hospitalier de Blois, Blois, France; 12 Centre Hospitalier Régional d’Orléans, Orléans, France; 13 AP-HP Pitié-Salpêtrière, Paris, France; 14 Polyclinique du Parc Drevon, Dijon, France; 15 Unicancer, Paris, France; Harvard Medical School, UNITED STATES

## Abstract

**Background:**

In patients treated with cardiotoxic chemotherapies, the presence of cardiovascular risk factors and previous cardiac disease have been strongly correlated to the onset of cardiotoxicity. The influence of overweight and obesity as risk factors in the development of treatment-related cardiotoxicity in breast cancer (BC) was recently suggested. However, due to meta-analysis design, it was not possible to take into account associated cardiac risk factors or other classic risk factors for anthracycline (antineoplastic antibiotic) and trastuzumab (monoclonal antibody) cardiotoxicity.

**Methods and findings:**

Using prospective data collected from 2012–2014 in the French national multicenter prospective CANTO (CANcer TOxicities) study of 26 French cancer centers, we aimed to examine the association of body mass index (BMI) and cardiotoxicity (defined as a reduction in left ventricular ejection fraction [LVEF] > 10 percentage points from baseline to LVEF < 50%). In total, 929 patients with stage I–III BC (mean age 52 ± 11 years, mean BMI 25.6 ± 5.1 kg/m^2^, 42% with 1 or more cardiovascular risk factors) treated with anthracycline (86% epirubicin, 7% doxorubicin) and/or trastuzumab (36%), with LVEF measurement at baseline and at least 1 assessment post-chemotherapy were eligible in this interim analysis. We analyzed associations between BMI and cardiotoxicity using multivariate logistic regression. At baseline, nearly 50% of the study population was overweight or obese. During a mean follow-up of 22 ± 2 months following treatment completion, cardiotoxicity occurred in 29 patients (3.2%). The obese group was more prone to cardiotoxicity than the normal-weight group (9/171 versus 8/466; *p* = 0.01). In multivariate analysis, obesity (odds ratio [OR] 3.02; 95% CI 1.10–8.25; *p* = 0.03) and administration of trastuzumab (OR 12.12; 95% CI 3.6–40.4; *p <* 0.001) were independently associated with cardiotoxicity. Selection bias and relatively short follow-up are potential limitations of this national multicenter observational cohort.

**Conclusions:**

In BC patients, obesity appears to be associated with an important increase in risk-related cardiotoxicity (CANTO, ClinicalTrials.gov registry ID: NCT01993498).

**Trial registration:**

ClinicalTrials.gov NCT01993498.

## Abstract 2

Charles Guenancia and colleagues investigate the association of obesity to breast cancer treatment related cardiotoxicity.

## Author summary

**Why was this study done?:**

- Anthracyclines remain a cornerstone of breast cancer therapy, in combination with new-generation targeted drugs such as trastuzumab, and represent the major culprit in chemotherapy-induced heart disease.
- In a recent meta-analysis, we showed that overweight and obesity increased cardiotoxicity, but it was not possible to take into account associated cardiac risk factors or other classic risk factors for anthracycline and trastuzumab cardiotoxicity.
- We examined the association of body mass index (BMI) and cardiotoxicity using prospective data collected in the CANTO study, a French national cohort.

**What did the researchers do and find?:**

- In patients treated for stage I–III breast cancer with anthracyclines and/or trastuzumab, being obese was associated with increased risk of developing cardiotoxicity, regardless of other predictors of cardiotoxicity.
- Among all the classic risk factors for cardiotoxicity, overweight and obesity were by far the most common risk factors in this breast cancer population.

**What do these findings mean?:**

- Obesity appears to be a risk factor for cardiotoxicity in breast cancer patients.
- Overweight and obese patients may benefit from careful cardiac screening and follow-up during and after chemotherapy.

## Introduction

Remarkable progress in the treatment of early breast cancer (stages I–IIIA), including multiple combinations of drugs, radiation therapy, and surgery, has been achieved in the past 2 decades. Nevertheless, anthracyclines remain a key element of breast cancer (BC) therapy in combination with new-generation targeted drugs such as trastuzumab, and represent an important cause of chemotherapy-induced heart disease [1].

Cardiotoxicity is a serious side effect of both agents, and its onset may even occur months to years after completion of cancer primary treatment [2,3]. Cardiotoxicity may severely impair the quality of life and overall survival of BC patients [3].

Cancer and cardiovascular disease (CVD) were previously considered 2 different pathologies. Recent data show that they share multiple risk factors, suggesting that there might be a common biological pathway [4]. A high body mass index (BMI) at diagnosis is often associated with a worse prognosis in BC [5,6]. Epidemiological studies show that obesity can increase the incidence of some BCs, lead to a poorer treatment outcome and quality of life after cancer diagnosis, and increase cancer-related mortality [7,8]. Recently, the influence of overweight and obesity as aggravating factors in the development of cardiotoxicity has been highlighted [9]. Animal models have suggested that overweight and obesity increase the risk of cardiotoxicity [10,11].

In a recent meta-analysis, we showed that overweight and obesity were risk factors for cardiotoxicity in treatment with anthracyclines and sequential anthracyclines and trastuzumab [9]. However, due to meta-analysis design, we could not consider associated cardiac risk factors or other classic risk factors for anthracycline and trastuzumab cardiotoxicity (older age, concomitant chemotherapy or previous radiation therapy, and having multiple cardiovascular risk factors such as smoking, hypertension, diabetes, and dyslipidemia).

Thus, using prospective data collected in the CANTO study, a French national cohort, we aimed to examine the association of BMI and cardiotoxicity.

## Methods

### Study design

OCATO (Obesity and CArdioTOxicity in breast cancer) was an ancillary study to the CANTO (CANcer TOxicities) trial [12]. CANTO is a French longitudinal multicenter cohort study designed to evaluate chronic toxicities in patients treated for non-metastatic BC. Patient recruitment and follow-up (from March 2012 to December 2014) was carried out in 26 French cancer centers. Patients were systematically assessed at diagnosis and at 3–6, 12, 36, 48, and 60 months after treatment completion, defined as end of chemotherapy, radiotherapy, or surgery. Trastuzumab and endocrine therapy could be ongoing. This study is reported as per the Strengthening the Reporting of Observational Studies in Epidemiology (STROBE) guidelines (S1 Checklist). This study is supported by the Unicancer group. All patients enrolled in the study provided written informed consent. Approval was obtained from French authorities (Agence française de sécurité sanitaire et des produits de santé: B111158-20) and ethics committees (Comité de protection des personnes: 11–039). An independent international advisory board was set up to guarantee the integrity of the study (ClinicalTrials.gov registry ID: NCT01993498; ID-RCB: 2011-A01095-36).

### Study population

CANTO patients met the following inclusion criteria: female patients aged 18 years and over covered by the national social security system, with histologically proven non-metastatic invasive BC (cT0 to cT3, CN0–3) and without previous cancer treatment.

**Fig 1:**
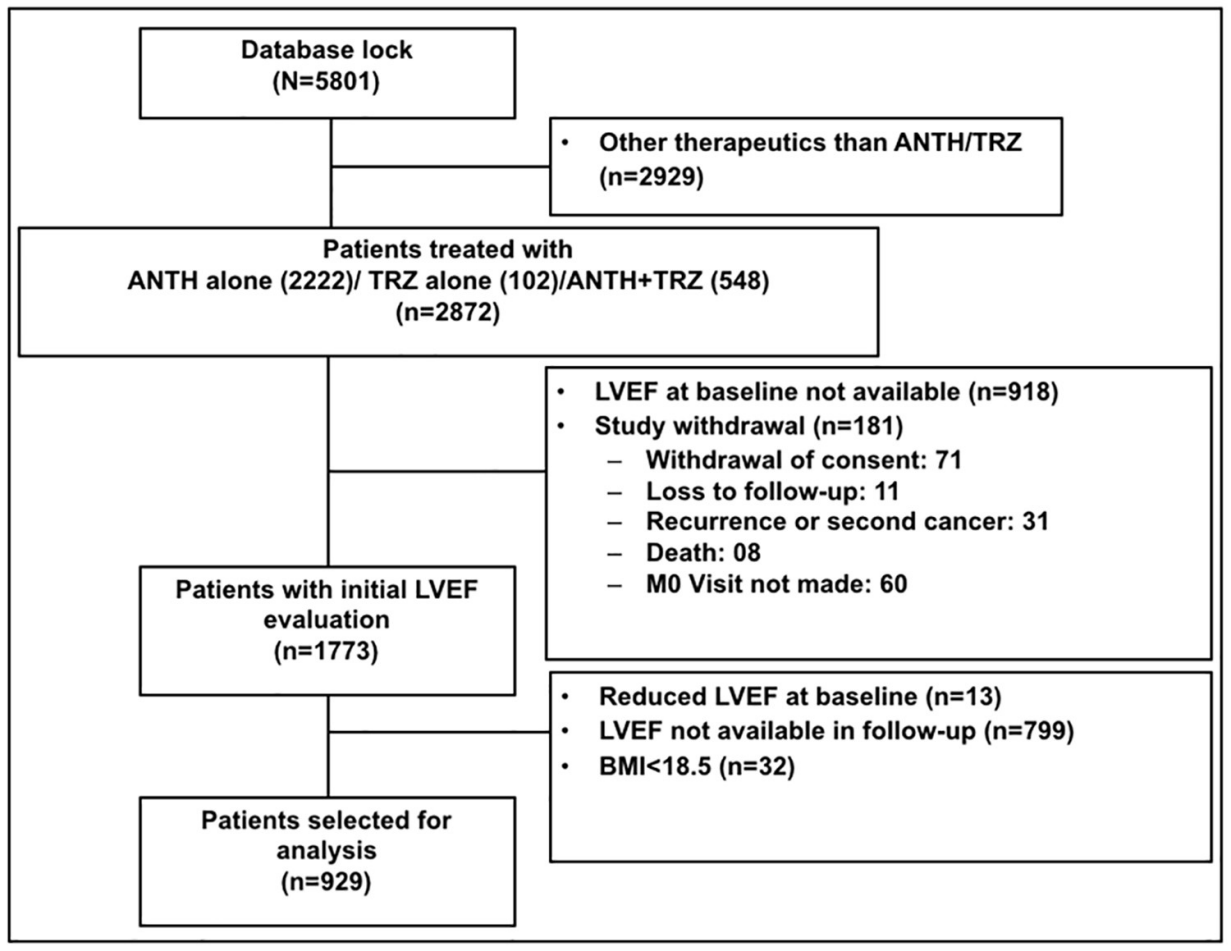
Flowchart. ANTH, anthracycline; LVEF, left ventricular ejection fraction; M0, month 0; TRZ, trastuzumab.

We accessed information at baseline, 3–6 months, and 12 months after the end of treatment from 5,801 women with data available in the most recent CANTO data lock (July 2018). For this interim analysis, we excluded patients treated with different therapies other than standard chemotherapy including anthracycline and/or trastuzumab (*n* = 2,929), patients with no left ventricular ejection fraction (LVEF) measurement at baseline (*n* = 918)or no available follow-up measurements (*n* = 799), patients with LVEF at baseline < 50% (*n* = 13), patients with BMI < 18.5 kg/m^2^ (*n* = 32), and patients with evidence of local, regional, or metastatic recurrence of BC (*n* = 181). Our final analytic cohort included 929 patients (Fig 1).

### Study outcomes

In the present study, the primary end point was occurrence of cardiotoxicity. Cardiotoxicity was defined in accordance with current guidelines as a reduction in LVEF > 10 percentage points from baseline to LVEF < 50% [13].

### Study covariates

Our independent variable was BMI at baseline, categorized following WHO as overweight (25 ≤ BMI < 30 kg/m^2^), obese (BMI ≥ 30 kg/m^2^), and normal (18.5 ≤ BMI < 25 kg/m^2^) [14].

As covariates we included demographic, clinical, and treatment variables, such as age at diagnosis; cardiovascular risk factors collected at baseline including previous hypertension, diabetes mellitus, dyslipidemia, and current smoking; dose of anthracycline; type of chemotherapy; and left-sided radiotherapy performed.

### Statistical analysis

Normal distribution of variables was verified using the Kolmogorov–Smirnov test. Normally distributed continuous data were expressed as mean values (±SD). Differences between groups were analyzed for statistical significance with the *t* test, χ^2^ test, or Fisher exact test as appropriate. Categorical variables were reported as percentages.

A multivariate regression analysis was used to explore the relationships between cardiotoxicity and BMI and baseline covariates. For this analysis, a forward stepwise procedure was used in the logistic regression to select significant factors, in which α (in) = 0.20 and α (out) = 0.10. A second multivariate logistic regression analysis was carried out after adjustment for known cardiovascular risk factors [15]. The odds ratios (ORs) and 95% confidence intervals (CIs) were estimated from regression coefficients. A *p*-value of <0.05 was considered statistically significant. All analyses were performed using the SAS statistical package (version 9.4; SAS Institute, Cary, NC).

## Results

We identified a total of 929 patients who met our inclusion criteria. The anthracycline regimens administered to patients included epirubicin (86%) and doxorubicin (7%). Trastuzumab was administered alone to 67 patients (7%) and combined with standard chemotherapy including anthracycline for 338 patients (36%).

Patients were predominantly younger than 65 years (87%), with a mean age of 52 ± 11 years (Table 1). In all, 394 (42%) patients had 1 or more cardiovascular risk factors. At baseline, nearly 50% of the study population was overweight or obese.

**Table 1:** Baseline characteristics.

Variable	Total (*N* = 929)	Cardiotoxicity (*n* = 29)	No cardiotoxicity (*n* = 900)	*p*-Value
Age (years)	52.4 ± 11.2	56.3 ± 10.9	52.2 ± 11.1	<0.001
Age > 65 years	125 (13.4%)	7 (24.1%)	118 (13.1%)	0.08
Age < 40 years	110 (11.8%)	2 (6.8%)	108 (12.0%)	0.56*
BMI (kg/m^2^)	25.6 ± 5.1	27.51 ± 5.3	25.6 ± 5.1	<0.001
BMI ≥ 25 kg/m^2^	463 (49.8%)	21 (72.4%)	442 (49.1%)	0.01
Cardiovascular risk factors				
Hypertension	187 (20.1%)	9 (31.0%)	178 (19.8%)	0.13
Diabetes mellitus	39 (4.2%)	2 (6.9%)	37 (4.1%)	0.34*
Dyslipidemia	92 (9.9%)	6 (20.7%)	86 (9.5%)	0.04
Smoking	172 (18.5%)	7 (24.1%)	165 (18.3%)	0.42
Coronary artery disease	2 (0.2%)	0 (0%)	2 (0.2%)	—
High cumulative dose of anthracycline				
Doxorubicin > 250 mg/m^2^	3 (0.3%)	0 (0%)	3 (0.3%)	—
Epirubicin > 600 mg/m^2^	0 (0%)	0 (0%)	0 (0%)	—
Chemotherapy agents				
Doxorubicin	62 (6.6%)	2 (6.9%)	60 (6.7%)	0.99*
Epirubicin	800 (86.1%)	22 (75.9%)	778 (86.4%)	0.10
Trastuzumab	405 (43.6%)	26 (89.7%)	379 (42.1%)	<0.001
Taxane	890 (95.8%)	28 (96.5%)	862 (95.8%)	0.99*
Alkylating agent	867 (93.3%)	28 (96.5%)	839 (93.2%)	0.71*
Left chest wall radiotherapy	484 (51.8%)	13 (44.8%)	471 (52.3%)	0.42
LVEF (%)	65.8 ± 6.3	61.2 ± 5.8	65.9 ± 6.2	<0.001
Diagnostic tool				0.72
Nuclear cardiac imaging (MUGA)	713 (76.9%)	20 (74.1%)	693 (77.0%)	
Echocardiography	214 (23.1%)	7 (25.9%)	207 (23.0%)	
Follow-up (months)	22.5 ± 2.2	21.3 ± 2.7	22.6 ± 2.2	<0.001

Data given as mean ± SD or number (percent). Missing data (*n*): diagnostic tool (2).

*Fisher exact test.

BMI, body mass index; LVEF, left ventricular ejection fraction; MUGA, multigated acquisition.

**Fig 2:**
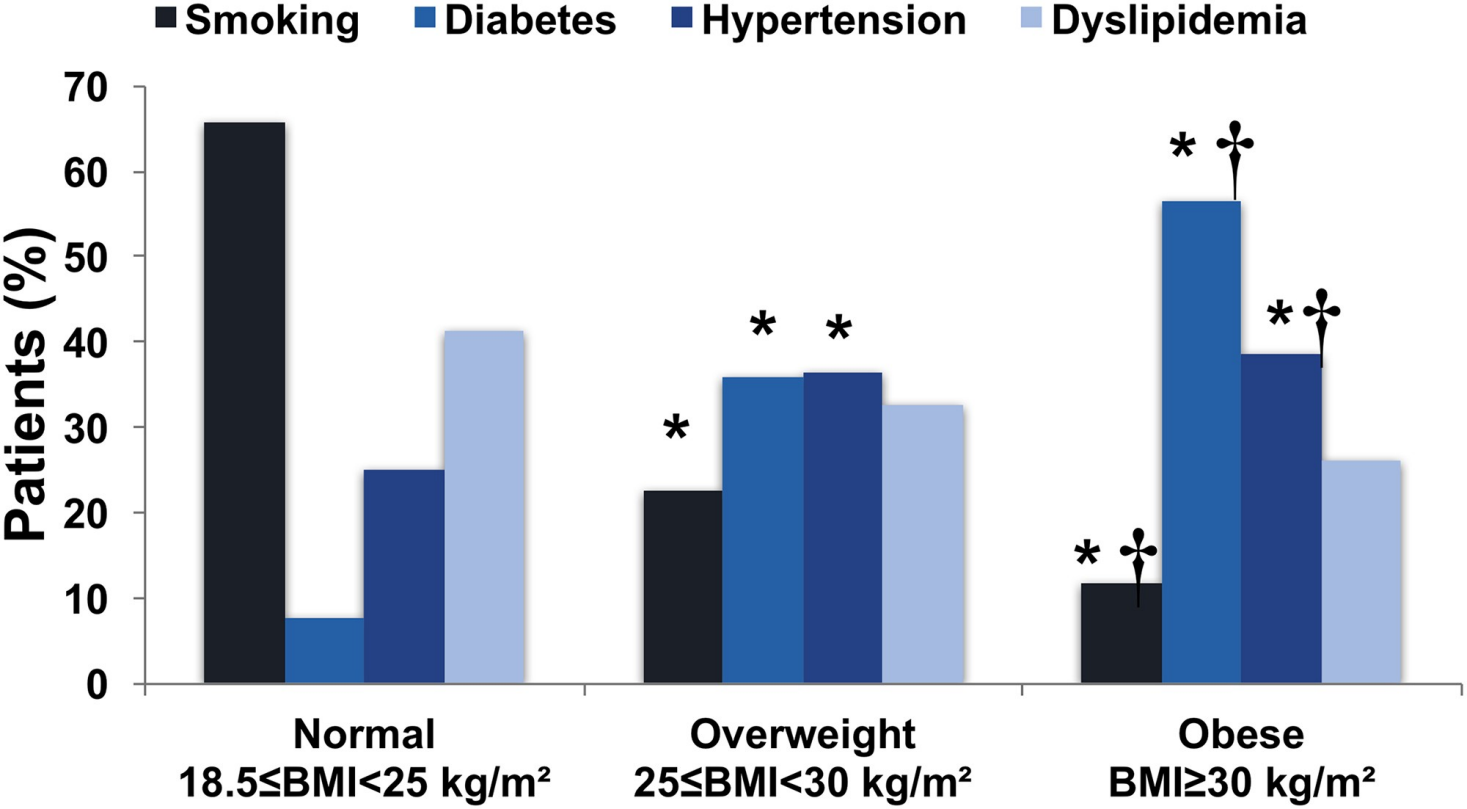
Distribution of cardiovascular risk factors according to body mass index category. *p < 0.05 versus normal weight; ^†^*p* < 0.05 versus overweight.

Higher BMI was associated with a higher prevalence of cardiovascular risk factors (Fig 2). Overweight and obese patients were more likely to have previous hypertension (*p <* 0.001) and diabetes (*p <* 0.001).

During a mean follow-up of 22 ± 2 months following treatment completion, cardiotoxicity occurred in 29 patients (3.2%), of whom 23 (73%) received anthracycline and trastuzumab, 5 (17%) received trastuzumab alone, and 3 (10%) received anthracycline alone. The preferred tool for cardiac function assessment was nuclear cardiac imaging by multigated acquisition (MUGA) (77%), with no difference regarding the diagnostic tool (MUGA or echocardiography) between the patients who developed cardiotoxicity and those who did not (Table 1).

**Fig 3:**
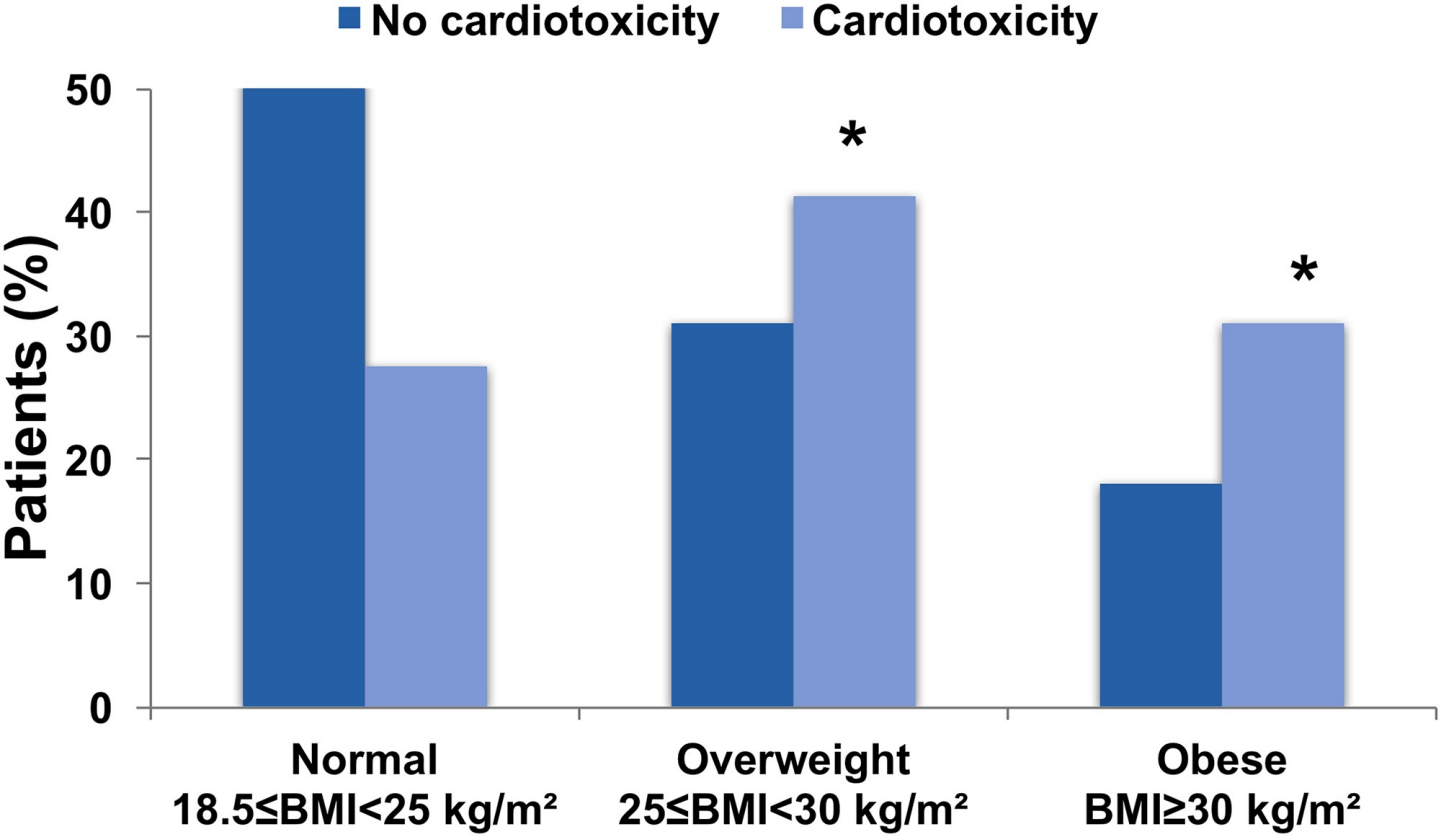
Association of body mass index with cardiotoxicity. *p < 0.05 versus normal weight.

BMI category distribution (*p* = 0.01) and mean age (*p* < 0.001) at baseline were significantly different between patients with and without cardiotoxicity. Compared with individuals in the normal-weight group (8/466), individuals in the overweight (12/292) and obese (9/171) groups were more likely to have cardiotoxicity (*p* = 0.01) (Fig 3). There was no significant difference in cardiotoxicity occurrence between overweight and obese group (*p* = 0.56).

In multivariate analysis, BMI ≥ 30 kg/m^2^ and trastuzumab administration were independently associated with cardiotoxicity (Table 2). When BMI category was included in a multivariate analysis adjusted for having multiple cardiovascular risk factors (≥2 risk factors among smoking, hypertension, diabetes, and dyslipidemia) and trastuzumab administration, obesity (OR 3.02; 95% CI 1.10–8.25; *p* = 0.03) but not overweight (OR 2.43; 95% CI 0.97–6.11) was independently associated with cardiotoxicity (Table 3).

**Table 2:** Univariate and multivariate analysis of the factors independently associated with cardiotoxicity.

Characteristics	Univariate analysis			Multivariate analysis		
	OR	95% CI	*p*-Value	OR	95% CI	*p*-Value
Age > 65 years	2.10	0.88–5.04	0.09			
BMI: overweight versus normal	2.45	0.99–6.07	0.05	2.47	0.99–6.20	0.05
BMI: obese versus normal	3.18	1.20–8.38	0.01	3.35	1.25–8.97	0.01
Hypertension	1.82	0.81–4.07	0.14			
Diabetes mellitus	1.72	0.39–7.54	0.46			
Dyslipidemia	2.46	0.97–6.23	0.05			
Smoking	1.41	0.59–3.37	0.43			
High cumulative dose of anthracycline	—	—	—			
Trastuzumab	11.91	3.58–39.65	<0.001	12.12	3.6–40.4	<0.001
Taxane	1.23	0.16–9.31	0.83			
Alkylating agent	2.03	0.27–15.21	0.48			
Left chest wall radiotherapy	0.74	0.35–1.55	0.42			

BMI, body mass index; OR, odds ratio.

**Table 3:** Multivariate analysis of the association between BMI category and cardiotoxicity.

Model	OR (95% CI)		
	Normal weight	Overweight	Obese
Model 1	1 (Reference)	2.45 (0.99–6.07)	3.18 (1.20–8.38)
Model 2	1 (Reference)	2.43 (0.98–6.03)	2.87 (1.07–7.74)
Model 3	1 (Reference)	2.43 (0.97–6.11)	3.02 (1.10–8.25)

Model performance (area under curve [AUC], Hosmer and Lemeshow goodness-of-fit test): Model 1: BMI category; AUC 0.64 (95% CI 0.56–0.74), χ^2^ 2.24, *p* = 0.69. Model 2: Model 1 + multiple cardiovascular risk factors (≥2 risk factors among smoking, hypertension, diabetes, and dyslipidemia); AUC 0.64 (95% CI 0.54–0.74), χ^2^ 0.17, *p* = 0.98. Model 3: Model 2 + trastuzumab; AUC 0.80 (95% CI 0.74–0.86), χ^2^ 2.04, *p* = 0.84.

## Discussion

In this large-scale prospective study, we found that in BC patients treated with anthracycline and/or trastuzumab, being obese was independently associated with a higher rate of cardiotoxicity compared to normal-weight patients.

In the literature, clinical decompensation occurs in 2%–4%, subclinical structural change in 9%–11%, and biomarker rise in 30%–35% of cancer chemotherapy patients [16], mainly depending on the total dose of anthracycline and on the criteria employed to define cardiotoxicity [17]. In our study, the incidence of cardiotoxicity was 3.2%. This low rate of cardiotoxicity could be explained by the definition used. Indeed, until recently, cardiotoxicity definitions were heterogeneous among the various studies published. The cardiac side effects of anthracycline and trastuzumab are mainly expressed by a decrease in LVEF [13]. Different cutoff values of LVEF have been previously used to define cardiac toxicity [18]. We followed a European Society of Cardiology position paper on cancer treatments and cardiovascular toxicity that defined cardiotoxicity as a drop of LVEF of at least 10 points to a LVEF < 50% [13]. Moreover, the CANTO study is in a modern setting of chemotherapy and mainly includes a young and healthy BC population. Considering that the classic risk factors of cardiotoxicity are older age, high anthracycline cumulative doses, and previous CVD, the relatively low rate of cardiotoxicity can thus be easily explained.

The incidence of cardiotoxicity after chemotherapy agents may be increased by smoking or preexisting CVD (or individual patient genetic predisposition to CVD) [13,19,20]. Data obtained in large cohorts show that patients who have survived cancer are significantly more prone to have cardiovascular risk factors such as overweight, hypertension, diabetes, and dyslipidemia compared with matched controls who have not had cancer [15]. Higher risk of CVD and CVD-related death in cancer survivors is probably due to multiple factors. It might involve cardiotoxic effects of different cancer treatments as well as comorbidities and unhealthy lifestyle habits [19].

In our study, cardiotoxicity was associated with cardiovascular risk factors such as age, dyslipidemia, and overweight/obesity. Interestingly, despite the absence of previously known CVDs, patients who developed cardiotoxicity had a lower (but normal) LVEF at the beginning of the chemotherapy. These results highlight the higher prevalence of cardiovascular risk factors in these patients and the need to carefully assess cardiological background before any cardiotoxic drug prescription. After multivariate analysis, among all cardiovascular risk factors, only obesity remained significantly associated with cardiotoxicity.

We found in this cohort that being obese was a major and an independent risk factor for cardiotoxicity. The epidemiological association between obesity and heart failure is well established. Kenchaiah et al. [21,22] described in 2 studies that every 1-kg/m^2^ increase in BMI was associated with an 11% increased risk of heart failure. Moreover, in these studies, overweight and obese patients had respectively a 49% and a 180% increased risk of developing heart failure at follow-up. In heart failure patients, obesity is an important clinical factor in both acute and long-term care. Obesity was associated with higher acute severity of heart failure in a dataset of 219,465 patients hospitalized for heart failure exacerbation [23].

Emerging evidence indicates a link between obesity and cardiotoxicity. We recently demonstrated in a rodent model submitted to postnatal overfeeding that overweight was associated with more severe cardiac alterations following the administration of anthracyclines and trastuzumab [11]. Therefore, we tested whether these results could be transposed to humans. We performed a random-effects and network meta-analysis including 15 studies and 8,745 patients with localized and metastatic BC, treated with adjuvant or neoadjuvant anthracyclines and/or trastuzumab [9]. We found that obesity/overweight was significantly associated with a higher risk of developing cardiotoxicity after anthracyclines and sequential anthracyclines and trastuzumab regimens. Associations were independent of study design, year of publication, drug regimen, and definitions of cardiotoxicity and overweight/obesity. However, we were unable to isolate the contribution of obesity-related cardiovascular risk factors, such as diabetes and hypertension, from that of obesity itself in this unadjusted analysis. The present study shows the association of obesity with cardiotoxicity in the early BC population. In this large scale, prospective, adjusted analysis, being obese was associated with a 3-fold increased risk of developing cardiotoxicity, regardless of other predictors of cardiotoxicity. Almost half of our patients had a BMI > 25 kg/m^2^, suggesting that overweight and obesity are commonly occurring risk factors for cardiotoxicity in patients with early BC.

Our results show that overweight and obese patients would certainly benefit from careful cardiac screening and follow-up during and after chemotherapy. Recently, several randomized studies showed a negative or modest effect of heart failure drugs such as beta blockers or angiotensin-renin system inhibitors in preventing cardiotoxicity [24]. In light of our results, we suggest that future studies should specifically focus on overweight and obese patients. Molecular pathways underlying the interaction between obesity and heart function, and leading to a higher probability of heart failure upon treatment with anthracyclines and trastuzumab, remain to be elucidated. The role of adipokines is considered to be at the intersection between obesity and CVDs, and adipokine dysregulation is a prominent hallmark of dysfunctional adipose tissue in obesity [25,26].

The current study has several limitations. One potential limitation is the relatively short follow-up of this interim analysis. With a mean follow-up of 22 months, we have likely captured the most likely timeframe for occurrence of early cardiotoxicity. In addition, centers measured LVEF using their preferred method, either echocardiography or MUGA. Nevertheless, variability could have been minimized if the same modality for repeated measurements had been required throughout the study. It was also not possible to study post-trastuzumab recovery in all patients due to missing data. Finally, many patients in the CANTO cohort had to be excluded due to lack of requisite baseline or follow-up LVEF measurements, leading to some selection bias.

### Conclusion

In conclusion, obese women with early stage BC treated with anthracyclines and/or trastuzumab may be at higher risk of experiencing drops in LVEF. Our results should encourage further research into the mechanisms linking obesity to cardiotoxicity, and personalized risk assessment for cardiac complications following cardiotoxic therapies could offer several potential benefits to patients.

## Supporting information

S1 Checklist STROBE checklist of the OCATO study. (DOCX)

## Abbreviations

BC: breast cancer
BMI: body mass index
CVD: cardiovascular disease
LVEF: left ventricular ejection fraction
MUGA: multigated acquisition
OR: odds ratio
